# New-onset diabetes as an emerging risk group for early detection of pancreatic cancer: current evidence, clinical challenge, and future directions

**DOI:** 10.3389/fgstr.2025.1645459

**Published:** 2026-01-12

**Authors:** Lan Valerie Tao, Joanna M. Karasinska, Vanessa G. P. Souza, Jonathan M. Loree, James D. Johnson, Daniel J. Renouf, David F. Schaeffer

**Affiliations:** 1 Department of Basic and Translational Research, British Columbia Cancer Research Institute, Vancouver, BC, Canada; 2 Interdisciplinary Oncology Program, University of British Columbia, Vancouver, BC, Canada; 3 Pancreas Centre British Columbia, Vancouver, BC, Canada; 4 Division of Medical Oncology, British Columbia Cancer, Vancouver, BC, Canada; 5 Department of Cellular and Physiological Sciences, Life Sciences Institute, University of British Columbia, Vancouver, BC, Canada; 6 Division of Anatomic Pathology, Vancouver General Hospital, Vancouver, BC, Canada; 7 Department of Pathology and Laboratory Medicine, University of British Columbia, Vancouver, BC, Canada

**Keywords:** biomarkers, diabetes, early detection of pancreatic cancer, new-onset diabetes, PDAC

## Abstract

Pancreatic ductal adenocarcinoma (PDAC) is a deadly disease characterized by late-stage manifestation and relative resistance to standard therapies. Challenges with early detection and a paucity of effective therapies lead to one of the lowest 5-year survival rates among all cancers. Individuals around 50 years and over presenting with new onset diabetes (NOD) have a higher risk for PDAC diagnosis within 3 years of diabetes onset compared to the rest of the population. In this review, we contextualize NOD within other types of diabetes presentations such as type 1 diabetes (T1D), type 2 diabetes (T2D), and type 3 diabetes (T3cD), unravel the bidirectional relationship between diabetes and PDAC, and highlight potential biomarkers that may distinguish PDAC-associated diabetes from other predominant types of diabetes. Although practical applications of NOD currently fall short from being clinically actionable, clinical trials are underway to stratify NOD patients with PDAC-associated diabetes. Ultimately, these efforts could offer the rationale to implement early detection screening strategies to this subgroup of PDAC patients.

## Introduction

Pancreatic ductal adenocarcinoma (PDAC) originates from the exocrine cells of the pancreas and accounts for 90% of all pancreatic cancers (1). PDAC is driven by oncogenic mutations in *KRAS*, found in 90% of cases, along with loss of function mutations in *TP53*, *SMAD4* and *CDKN2A* (2). Early-stage disease is typically asymptomatic; however, as the tumor progresses, patients commonly present with weight loss, abdominal pain, jaundice, and other systemic manifestations (3). A major contributor to the profound weight loss observed in PDAC is pancreatic exocrine insufficiency (PEI), which arises when tumor-related obstruction or parenchymal destruction impairs the secretion of digestive enzymes. As a result, patients experience maldigestion and progressive malnutrition, which worsen quality of life and accelerate cachexia, a leading cause of morbidity and mortality in PDAC (4–6).

PDAC has a 5-year survival rate of 13% (7), attributed to the lack of effective early detection and advanced disease presentation where the feasibility of curative surgery is limited (8). Late-stage diagnosis results in a drop in 5-year survival rate from 44% to 3% (7). Early detection using imaging, family history, physical examination, cell cytology, tissue biopsies and blood-based biomarkers have proved successful for cancers of the breast, colorectum, prostate, endometrium, ovarian, and lung (9). Circulating CA-19.9 antigen is the primary biomarker for diagnosing and monitoring PDAC in symptomatic individuals but falls short as a routine screening tool due to the influence of various benign conditions (10). An optimal PDAC detection tool should possess high sensitivity and specificity to balance out accurate detection in affected individuals and the risk posed to the greater population of individuals that will likely not develop the disease (11).

There has been a shift in focus to direct cancer surveillance to high-risk subgroups (11), including individuals with familial hereditary risk, presence of precursor lesions like pancreatic intraepithelial neoplasia (PanIN) and intraductal papillary mucinous neoplasia, and chronic pancreatitis (11). One emerging high-risk group that has generated interest in the early detection of PDAC are individuals that present with new-onset diabetes (NOD). Although there is no standardized definition of NOD and its adjacent terminology “recent-onset diabetes”, these terminologies were widely used to describe individuals who recently present with diabetes (12). The association between NOD and PDAC was introduced in earlier comparative studies (13), while the first quantitative analysis of NOD and PDAC risk group originates from the observation that approximately 1% of NOD individuals aged 50 and older, who have had diabetes for <3 years, are nearly eight times more likely to develop PDAC compared to the general population (14). The association between NOD and PDAC was further reported in studies conducted within diverse patient populations (15–27). Therefore, NOD has transformed from a clinical symptom to an emerging risk group for PDAC that requires further evaluation to determine its applicability in clinical settings.

**Figure 1:**
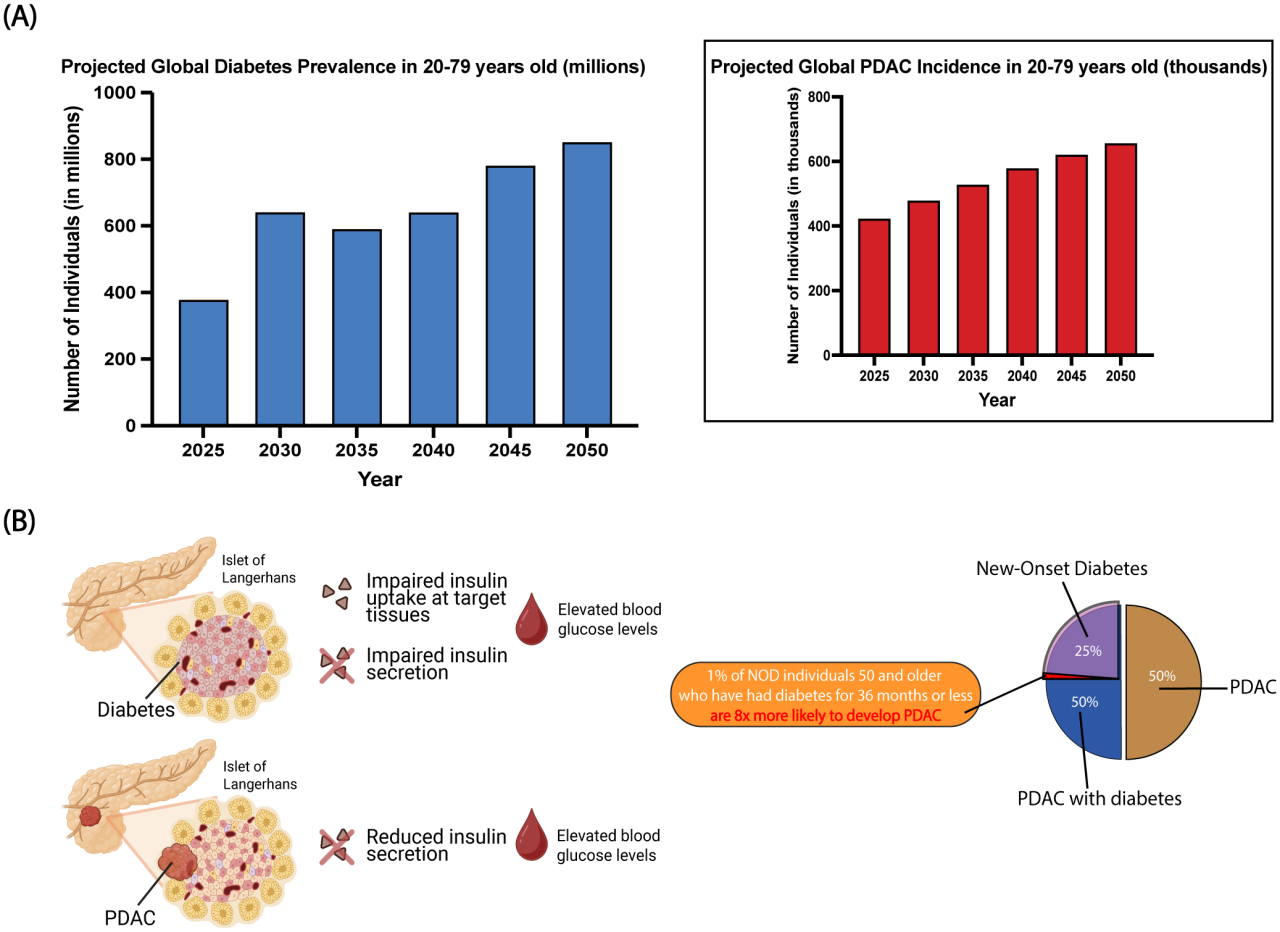
Trends in diabetes and PDAC epidemiology and their intersecting mechanism of action. **(A)** The concurrent increase in the projected global diabetes prevalence and PDAC incidence in 20–79-year-olds aligns with their known epidemiological association. **(B)** Both diseases can result in an increase in blood glucose levels as a cause of islet disruption and insulin dysfunction. Epidemiological data show that 50% of PDAC patients simultaneously have diabetes at the time of diagnosis. Half of those individuals are predicted to have NOD wherein 1% of NOD individuals have an increased PDAC risk.

With global PDAC incidence rising alongside diabetes prevalence in 20–79-year-old individuals from 2025 to 2050, it prompts the question as to how many new PDAC cases are linked to diabetes (**Figure 1A**) (28, 29). Without further interrogation, high blood glucose could be a presentation of diabetes or a symptom of PDAC-associated diabetes (**Figure 1B**). However, epidemiological data have indicated that approximately 50% of PDAC patients have diabetes at the time of diagnosis (**Figure 1B**). Half of those are likely NOD candidates with 1% of NOD individuals at a higher risk for PDAC diagnosis (**Figure 1B**) (30). In the context of hundreds of thousands of PDAC cases, 1% could account for thousands of patients facing a life-altering diagnosis of PDAC who may benefit from earlier surveillance within a 3-year window to prevent or delay disease progression.

Although it is well established that a small proportion of patients with NOD have an increased risk of subsequently being diagnosed with PDAC, translating this association into a broadly applicable clinical strategy remains challenging. NOD itself is a recognized cardiovascular and metabolic risk factor and, in certain subgroups, may represent an early warning sign of serious underlying disease, including PDAC (31–34). Despite long-standing recognition of the public health need to systematically evaluate NOD in the general population, this goal has not been achieved. Key barriers include the lack of standardized definitions, the biological and etiological heterogeneity of NOD, limited tools to distinguish types of diabetes at presentation, and uncertainties regarding cost-effectiveness and downstream clinical management in largely asymptomatic populations (34–45).

At the same time, PDAC remains intrinsically difficult to study due to its biological complexity, low incidence, and clinically silent progression until advanced stages, which limits early detection efforts. Together, these challenges highlight the need for improved strategies to identify individuals with NOD who are most likely to harbor occult PDAC and may benefit from targeted surveillance.

In this context, this review summarizes current knowledge of pancreatic cancer-associated NOD and highlights emerging biomarkers and clinical identifiers to distinguish PDAC-associated diabetes from more common types of diabetes, while also discussing ongoing clinical trials and the opportunities and limitations of NOD-based early detection strategies.

## Types of diabetes and PDAC risk

**Figure 2:**
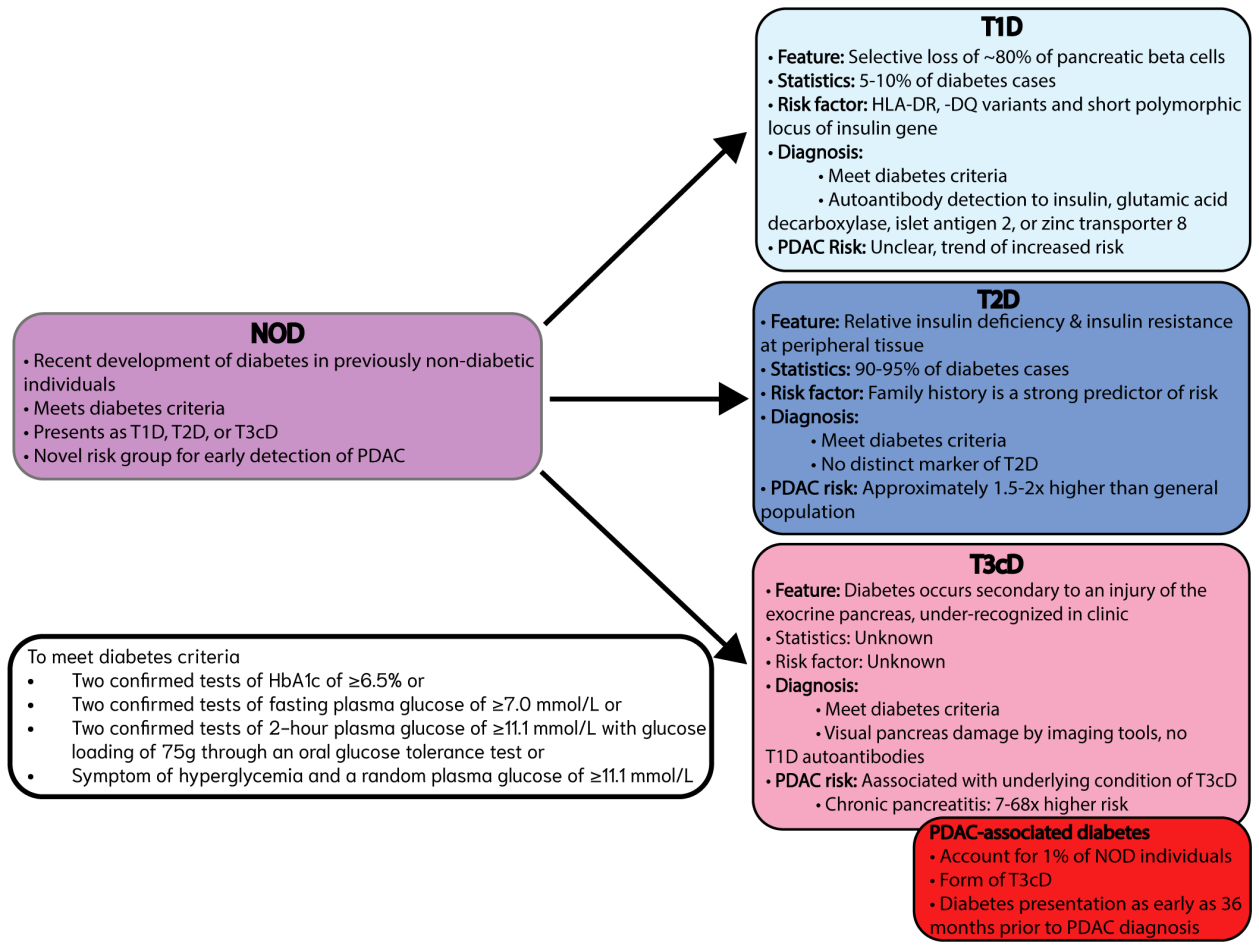
Framing PDAC-associated diabetes stemming from NOD within the broader context of different types of diabetes. Distinct features, statistics, risk factors, diagnostic criteria, and PDAC risk outlined for T1D, T2D and T3cD with PDAC-associated diabetes emerging as a distinct form of T3cD most relevant to NOD as a novel risk group for the early detection of PDAC. All diabetes types must meet diabetes criteria.

Diabetes describes a collection of metabolic disorders that disrupts the body’s ability to regulate glucose levels due to dysfunction in insulin secretion by pancreatic β-cells, uptake of insulin at insulin-dependent tissues, or both (46). A diabetes diagnosis relies on the clinical demonstration of increased glucose in the plasma or hemoglobin A1c (HbA1c) coating on red blood cells. Both can be detected by a minimally invasive blood draw by directly measuring HbA1c levels, fasting plasma glucose levels or an oral glucose tolerance test. To be clinically diagnosed with diabetes, an individual must meet the criteria with two consecutive confirmations of an HbA1c of 6.5% or greater, a fasting plasma glucose of ≥ 7.0 mmol/L, 2-hour plasma glucose of ≥11.1 mmol/L with glucose loading of 75g through an oral glucose tolerance test, or one definitive confirmation of hyperglycemia status and a random plasma glucose of ≥11.1 mmol/L (**Figure 2**) (46).

Type 1 diabetes mellitus (T1D) is an autoimmune disorder that accounts for 5-10% of cases of diabetes (47). Previously, T1D was considered primarily as a disease affecting children but it is now appreciated that T1D can occur at any age (**Figure 2**) (48). The pathogenesis of T1D involves the selective loss of up to 80% of pancreatic β-cells due to autoimmunity such that exogenous insulin treatment is required (49). Common genetic risks of T1D are attributed to human leukocyte antigen (HLA) -DR and -DQ variants and a short polymorphic locus linked to the insulin gene (50). Unique to the clinical diagnosis of T1D is the detection of autoimmune markers for autoantibodies to insulin, glutamic acid decarboxylase (GADA), islet antigen 2, or zinc transporter 8 in addition to elevated blood glucose levels (46). There are inconsistent reports on the associated bidirectional risk between T1D and PDAC with some studies reporting trends of increased PDAC risk associated with T1D (**Figure 2**) (51, 52).

Type 2 diabetes mellitus (T2D) accounts for 90-95% of all diabetes that occurs due to relative insulin deficiency and insulin resistance at peripheral tissues, resulting in chronically elevated blood glucose levels (53). The incidence of T2D increases with age and peaks between 55–59 years of age with family history as a strong predictor of risk (54). Pancreatic cancer risk was increased by 50% in individuals with T2D diagnosis of less than 4 years compared to individuals with T2D for over 5 years (55). The overall PDAC risk for T2D is approximately 1.5 to 2 times higher than that of the general population (**Figure 2**) (17, 30). Altogether, the complex interplay of pathologies linked to T2D contribute to an increased risk of PDAC development.

Type 3c Diabetes (T3cD), also known as pancreatogenic diabetes occurs secondary to an injury of the exocrine pancreas that results in the loss of insulin secretion for glucose homeostasis (56), including pancreatitis, hereditary haemochromatosis, cystic fibrosis, pancreatic cancer, genetic disorders, trauma, or pancreatectomy (57). T3cD does not have an associated risk for PDAC as it broadly categorizes multiple underlying conditions, however, chronic pancreatitis has been documented to have associated pancreatic cancer risk ranging as high as seven to sixty-eight times (**Figure 2**) (58). The key diagnostic T3cD features are meeting the standard diabetes criteria, visualizing pancreas damage by imaging tools, and the absence of T1D autoantibodies (56). Additionally, a distinguishing factor of T3cD presentation is the loss of glucagon secretion alongside insulin secretion loss (46, 56). As T3cD encompasses a variety of conditions and remains understudied and underdiagnosed its incidence is unclear (57). However, chronic pancreatitis has been studied in-depth due to its relevance as a high-risk group in pancreatic cancer, often used to represent incidence of T3cD, wherein chronic pancreatitis increases with age and peaks around 50–60 years of age (59). Current knowledge of PDAC risk is predominantly associated with T3cD originating from chronic pancreatitis, but PDAC risk driven by other T3cD-associated conditions lack clarity due to our poor understanding of T3cD (**Figure 2**).

NOD represents a subgroup of patients that encompass all recent diagnoses of diabetes that include T1D, T2D, and T3cD (**Figure 2**). In the new categorization of NOD as a high-risk group for PDAC, individuals with NOD stemming from PDAC-associated diabetes rather than other types of diabetes become particularly interesting as diabetes mediated by PDAC has the potential to enable early, non-invasive screening or prompt imaging and invasive diagnostic strategies (14). Current evidence for clinical application is insufficient, but ongoing research efforts are honing in on distinguishing clinical features and biomarkers of PDAC-associated diabetes to better capture this small, but important subgroup of patients in the early detection setting (**Figure 2**).

## Proposed molecular mechanisms of the bidirectional relationship of diabetes and PDAC

Although the association between diabetes and pancreatic cancer is emphasized by the high diabetes prevalence among PDAC patients over other cancers and noncancer controls (60), the mechanistic understanding remains complex, owing to the bidirectional effects of two highly linked diseases dysregulated in many processes and the discourse on the primary event in the pathogenesis of dysglycaemia (61, 62). Recent evidence has reignited the reconsideration of the established dogma that β-cell dysfunction precedes hyperinsulinemia as some studies demonstrate hyperinsulinemia as an early, common event resulting from β-cell dysfunction that leads to obesity and insulin resistance to initiate β-cell exhaustion, ultimately leading to diabetes presentation (62). As such, there is no clear resolution in the sequence of events in the pathogenesis of diabetes due to the opposing models, which not only challenge our mechanistic understanding of diabetes, but also by association, the mechanism of PDAC-associated diabetes.

In the context of diabetes as a risk factor for PDAC, proposed mechanisms broadly centralize around excessive lipid availability, activation of signaling molecules, and the dysregulation of glucose and insulin that helps promote PDAC (63–75).

**Figure 3:**
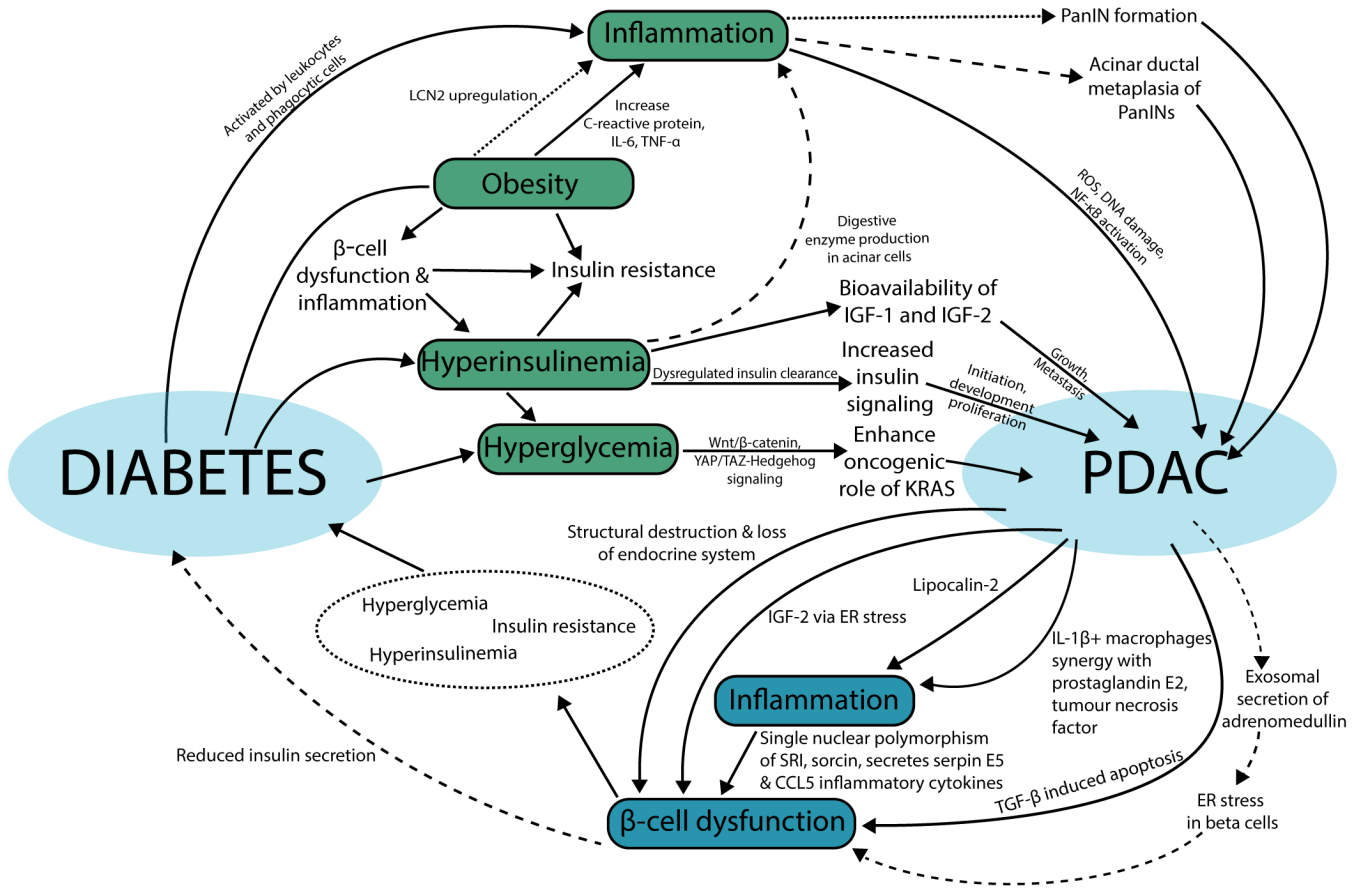
Mapping proposed mechanisms of the bidirectional relationship of diabetes and PDAC. On the top half, the relationship between diabetes to PDAC highlights key modulators in inflammation, obesity, hyperinsulinemia, and hyperglycemia, while the bottom half showcases inflammation and beta cell dysfunction as major modulators driving PDAC-associated diabetes. The size of the dashed lines indicates connected concepts. This bidirectional model represents the complex interplay between diabetes and PDAC.

Obesity is the leading risk factor for T2D and is a complex modulator of PDAC development as the pathophysiological features are a combination of excess lipids (63), inflammation (64), hyperinsulinemia, insulin resistance, and β-cell dysfunction (65), many of which are direct or indirect modulators too. The presence of excessive lipid buildup leading to enhanced production of adipose tissue-derived mediators and factors can promote inflammation (66). Specifically, markers of inflammation such as C-reactive protein, interleukin-6 (IL-6) and TNF-α have been found to be secreted by adipose tissue and immune cells like macrophages (**Figure 3**) (67). Moreover, an adipokine, LCN2, has been identified to be elevated in obese individuals to promote PanIN formation, increase inflammation and fibrosis in pancreatic cancer mouse models on a high fat diet (**Figure 3**) (68). Not only do adipose tissue-derived factors cause inflammation that can lead to pancreatic cancer, but the dysregulation of the factors themselves is associated with pancreatic cancer (**Figure 3**) (69).

Hyperglycemia, a condition defined by high levels of glucose in the blood is highly associated with T2D (53), drives the oncogenic role of KRAS in PDAC and enhances PDAC initiation and progression through Wnt/β-catenin signaling and Yes-associated protein (YAP) and transcriptional co-activator with PDZ-binding motif (TAZ)-Hedgehog signaling (**Figure 3**) (70–72). Hyperinsulinemia precedes hyperglycemia in the pathogenesis of T2D. Hyperinsulinemia leads to increased insulin signaling through the insulin receptor that could promote survival, growth, and proliferation through phosphoinositide 3-kinase (PI3K) and mitogen-activated protein kinase (MAPK) signaling in PDAC development and progression (**Figure 3**) (73). In a mouse model of PDAC initiation, hyperinsulinemia triggered insulin receptor signaling in acinar cells to enhance digestive enzyme production and inflammation leading to increased acinar to ductal metaplasia of PanIN precancerous lesions (**Figure 3**) (74). Similarly, hyperinsulinemia drives the bioavailability of insulin-like growth factor 1 (IGF-1) and 2 (IGF-2) (76). IGF-1 and IGF-2 are structurally similar to insulin and promote PDAC growth, metastasis, and therapeutic resistance (**Figure 3**) (77).

While the mechanistic understanding of diabetes as a risk factor for PDAC has been reinforced by association studies and PDAC initiation models (63–66, 68–71, 73, 74), investigation in PDAC-associated diabetes has been hindered by the lack of animal models. The recognition of diabetes and hyperglycemia resolution post-pancreatectomy in a proportion of pancreatic cancer patients established that diabetes may be induced by PDAC (75, 78).

Mechanisms underlying PDAC-associated diabetes centre around inflammation and β-cell dysfunction (79, 80). As PDAC flourishes and overtakes the pancreas parenchyma, the pancreatic endocrine system is prone to destruction in the form of β-cell dysfunction and loss (**Figure 3**) (81, 82), with clinical evidence of remnant pancreas volume loss leading to β-cell dysfunction as diabetes status progressed from normal, prediabetic to NOD secondary to PDAC (83). PDAC cell-secreted adrenomedullin within exosomes led to endoplasmic reticulum (ER) stress in β-cells resulting in dysfunction and ultimately decreased insulin secretion (80), while PDAC mouse models demonstrated that the depletion of β-cells is due to an increase in TGF β signaling that induces apoptosis of the β-cells resulting in diabetes (**Figure 3**) (81). Similarly, IGF-2 has been shown to induce β-cell dysfunction caused by ER stress (84). Although we may further infer the roles of insulin resistance and hyperglycemia in the progression from β-cell dysfunction to diabetes, the exact directionality of these two conditions have not been fully elucidated in the context of PDAC-associated diabetes (**Figure 3**).

Cytokine-induced inflammation is a common pathophysiological event in T2D in white adipose tissue capable of driving β cell inflammation and failure, insulin resistance, and other metabolic disorders (85). The activation of inflammation by leukocytes and phagocytic cells can trigger an intrinsic production of reactive oxygen species to cause unwanted DNA damage and activation of nuclear factor-kappaB (NF-κB), central players in cancer development, offering a potential mechanism of PDAC development through T2D-linked inflammation, although not exclusive to T2D (**Figure 3**) (64, 86). In parallel, inflammation is also an effect of the PDAC tumor microenvironment. The role of inflammation in PDAC-associated diabetes is compounded by the intricacy of inflammation and its natural role in the inflammatory response that range from harmless, harmful, to helpful aetiologies by activating an abundance of cytokines and cellular pathways (87). Within the PDAC microenvironment interleukin-1 beta (IL-1β) positive macrophages, prostaglandin E2, tumor necrosis factor, and lipocalin-2 can induce pro-inflammatory conditions (**Figure 3**) (68, 88). The effects of inflammation in the tumor microenvironment further β-cell dysfunction that ultimately lead to conditions like insulin resistance, hyperglycemia state and diabetes status (**Figure 3**) (85). A polymorphism in *SRI* that codes for sorcin, associated with the secretion of serpin E5 and CCL5 inflammatory cytokines, also triggers β-cell dysfunction (**Figure 3**) (89).

This section highlights the contribution of many modulators central to the relationship between diabetes to PDAC and vice versa with multiple proposed pathways and dysregulated processes that form a feedback loop that continuously exacerbates the effect of another modulator. Although multiple proposed mechanisms have been described, other complex mechanisms are likely involved that have not been discerned at this time.

## Clinical measures of NOD in pancreatic cancer

PDAC-associated diabetes within NOD individuals was first identified in a retrospective population-based study of 2, 122 Olmsted residents aged 50 years and over who met the diabetes criteria based on the National Diabetes Data Group’s recommendation of glycemic levels that was collected from 1950 to 1994. Longitudinal monitoring of patient medical records through the Rochester Epidemiology Project revealed that 0.85% of diabetes subjects developed PDAC within a 3-year period from diabetes onset, with an observed-to-expected ratio of 7.94 (95% CI, 4.70-12.55) compared to the general population (14). Follow-up studies in other cohorts indicated that the percentage of PDAC-associated diabetes originating from NOD range from 0.25-3% (15, 18, 21, 23, 24, 27). Other independent studies confirmed a higher risk score for PDAC in the PDAC-associated diabetes population compared to the T2D population (16, 17, 22, 25).

**Figure 4:**
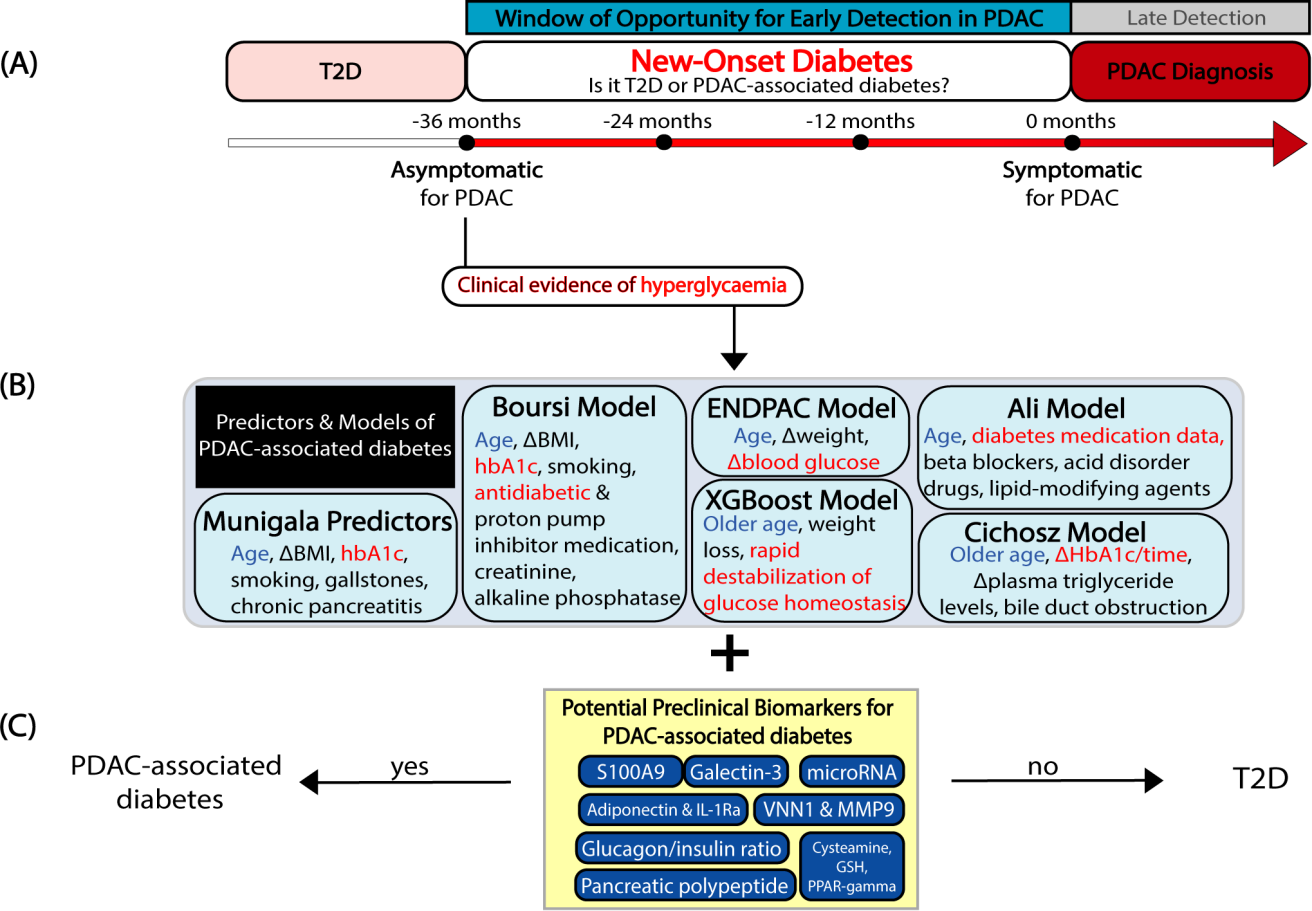
**(A)** Schematic representing a clinical window of opportunity 3 years preceding PDAC diagnosis for **(B)** the implementation of various predictors, models, **(C)** and biomarkers proposed in preclinical studies to help distinguish PDAC-associated diabetes from T2D in New-Onset Diabetes individuals.

Across all NOD studies highlighted, age and diabetes status are clinical measures relevant in the risk assessment of PDAC-associated diabetes (14–18, 21–27, 90). Most studies concluded that the peak age group of PDAC-associated diabetes within the NOD population is among older individuals 50 years and older, with the exception of one NOD study cutoff of 40 years and older (15, 19, 24, 25). Conversely, a couple of studies have proposed that younger NOD individuals had a greater propensity for PDAC (17, 90). A greater proportion of PDAC-associated diabetes in NOD individuals occur within 0 to 12, 12 to 24, and 24 to 36 months prior to PDAC diagnosis, while similar proportions of PDAC-associated diabetes and control populations occur past 36 months, thus establishing the criticality of diabetes presentation 3 years before PDAC as a window of opportunity for early detection (**Figure 4A**) (91). Additionally, patients are first noted to be hyperglycemic 3 years prior to diagnosis and diabetic 6 months prior to clinical diabetes diagnosis (92), suggesting a rapid change in glucose homeostasis (19, 93). Although multiple groups defined NOD presentation within this 3-year timeframe (15, 16, 23, 24), many studies defined NOD within a shorter timeframe of 2 years (18, 21, 22, 90), potentially narrowing the window of opportunity for early intervention.

Another risk factor in PDAC-associated diabetes is the presence of weight loss (18–20, 26, 93). It was demonstrated that ≥10% weight loss at PDAC diagnosis compared to weight prior to diabetes onset had a higher adjusted odds ratio in PDAC-associated diabetes individuals compared to T2D (93), while age-adjusted hazard risk for PDAC-associated diabetes increased with increasing weight loss in NOD individuals compared to T2D (26). This is further supported by another study that indicated that individuals 65 and older with weight loss greater than 2kg with no prior history of T2D may be indicative of NOD (20). These observations suggest that weight loss may be another critical determinant of risk for PDAC-associated diabetes in NOD individuals 2–3 years prior to PDAC diagnosis. Further work is required to establish the degree of weight loss most relevant in the identification of PDAC-associated diabetes within the NOD population.

## Predictors and models of new-onset diabetes for pancreatic cancer

To identify PDAC-associated diabetes among other NOD individuals, a PDAC-associated diabetes risk prediction model must be established based on measurable clinical parameters. The Munigala model discovered age, body mass index (BMI), smoking, chronic pancreatitis and gallstones as major predictors of PDAC in the NOD population up to 3 years before PDAC diagnosis in individuals 40 years of age and older (**Figure 4B**) (24). The Boursi multivariable model was created using the three most highly reported parameters of age, weight change in the form of BMI change per year, HbA1c along with additional variables of smoking, use of antidiabetic and proton pump inhibitor medications, creatinine and alkaline phosphatase within 3 years of PDAC diagnosis (**Figure 4B**) (23). This model was later validated by an independent group indicating that the Boursi model enriches PDAC-associated diabetes within the NOD population (94).

The Enriching New-Onset Diabetes for Pancreatic Cancer (ENDPAC) model was introduced to enrich and stratify NOD individuals based on their risk of PDAC development by including age of onset, weight change, blood glucose categories established by the American Diabetes Association that records blood glucose change a year before and at the time of NOD diagnosis (**Figure 4B**) (95). An ENDPAC score of ≥3 indicates high risk for PDAC development that warrants additional screening by CT imaging, endoscopic ultrasound, and further monitoring in 6 months (95). Currently, the model lacks definitive categorization of ENDPAC scores of 1-2. The need for further investigations in biomarkers has been recognized to better stratify these risk groups (**Figure 4B**) (95). This model was further validated by multiple research groups in independent cohorts (96, 97).

The Ali Model demonstrated that age and use of diabetes medication best discriminate PDAC-associated diabetes in a study conducted exclusively in women, with some predictive effects of beta-blockers, lipid-modifying agents, and acid disorder drugs (**Figure 4B**) (15). The Cichosz model uses age, rate of change in HbA1c levels, changes in plasma triglyceride levels and bile duct obstruction (**Figure 4B**) (98), while the XGBoost model uses weight loss, and rapid destabilization of glucose homeostasis as discriminating risk factors for PDAC-associated diabetes (**Figure 4B**) (19). Overall, these modeling studies highlight potential clinical parameters that require further prospective investigation to narrow down which of them best capture PDAC-associated diabetes in clinical settings.

## Potential biomarkers and identifiers of new-onset diabetes to distinguish PDAC-associated diabetes and T2D

Apart from clinical markers of T1D and clinical evidence of PDAC-associated diabetes that distinguish from T2D within NOD populations, there is a need to establish clinically relevant and reliable non-invasive blood biomarkers for the differentiation of diabetes and further knowledge in independent fields of T2D and PDAC. Metabolomics studies of T2D alone have highlighted alterations in branched-chain amino acids like isoleucine, leucine and valine, along with alterations in various lipids, sugars, and organic acids (99). Meanwhile, metabolomics study comparing T3cD secondary to chronic pancreatitis compared to T2D revealed an enrichment of bile acid biosynthesis, fatty acid biosynthesis, and sphingolipid metabolic pathways (100, 101). Although these metabolic studies do not elucidate biomarker difference of PDAC-associated diabetes and T2D specifically, there is an essentiality to understand outstanding differences in T2D and other T3cD to clearly establish unique biomarkers of PDAC-associated diabetes.

Current established evidence for PDAC-associated diabetes and T2D differentiation broadly spans a range of blood and tissue biomarkers involving microRNA, protein, and hormones that are directly or indirectly linked to inflammatory properties, gene regulation, glucose metabolism, or the insulin system. A panel of six serum microRNAs, miR-483-5p, miR-19a, miR-29a, miR-20a, miR-24, and miR-25 were shown to potentially discern PDAC-associated diabetes from T2D with an area under the receiver operating characteristic curve (AUC) of 0.902 (Table.1)(**Figure 4C**) (102). Moreover, Liao et al. combined high-throughput proteomics and bioinformatics in a PDAC cell line, an *in vitro* glucose uptake assay, and validation in blood serum in NOD patient samples that indicated a 10-fold higher transcriptomic expression in Galectin-3 and S100A9, two insulin resistance mediators, that distinguish PDAC-associated diabetes from T2D. Galectin-3 has an AUC of 0.83 with 72.1% sensitivity and 86.1% specificity, while S100A9 has an AUC of 0.77 with 69.8% sensitivity and 58.1% specificity (Table.1)(**Figure 4C**) (103). Meanwhile, Oldfield and colleagues combined mass-spectrometry, immunoassay and a literature-based search to identify higher concurrent levels of adiponectin, a hormone secreted by adipose tissue, and interleukin-1 receptor antagonist (IL-1Ra), a secreted anti-inflammatory protein. Circulating levels of adiponectin correlate with lower subcutaneous adipose tissue and visceral adipose tissue, while circulating IL-1Ra correlates with insulin resistance. Together, adiponectin and IL-1Ra have an AUC of 0.91 with an optimal sensitivity and specificity of 83.7% and 100%, respectively (**Table 1**) (**Figure 4C**) (104). In contrast, it was demonstrated that PDAC-associated diabetes patients have reduced postprandial pancreatic polypeptide response as measured by a lower incremental AUC compared to chronic pancreatitis associated diabetes, while higher pancreatic polypeptide was associated with T2D (Table.1)(**Figure 4C**) (105). Meanwhile, the glucagon/insulin ratio was elevated in PDAC-associated diabetes compared to T2D as a result of greater area of glucagon expressing cells in the islets. At a ratio of 7.4 ng/mU, the sensitivity and specificity of glucagon/insulin ratio was 77% and 69%, respectively (106). Furthermore, a clinical study showed that vanin-1 (VNN1) and matrix metalloproteinase 9 (MMP9) in the peripheral blood differentiated PDAC-associated diabetes from T2D with an AUC of 0.95, sensitivity of 95.83 and specificity of 76% (**Table 1**) (**Figure 4C**) (107). VNN1 is a pantetheinase anchor upstream of cysteamine, a key regulator of essential metabolic pathways, while MMP9 is a proteolytic enzyme linked to tissue remodeling, tumor weight and metastasis. Follow-up laboratory studies demonstrated that increased serum cysteamine, decreased glutathione (GSH) and PPAR-γ, downstream molecules of VNN1, were also associated with PDAC-associated diabetes. The AUC for all cysteamine, GSH, and PPAR-γ were 0.844, 0.863, and 0.826. Altogether, VNN1, cysteamine, GSH, and PPAR-gamma were shown to increase oxidative stress leading to islet dysfunction (**Table 1**) (**Figure 4C**) (108). These studies demonstrate the range of potential biomarkers that discriminate PDAC-associated diabetes from T2D. However, many of these biomarkers are not yet clinically useful, and further work is required to identify and refine the most reliable candidates, especially those demonstrating consistently high sensitivity and specificity across multiple independent validation cohorts. Continued evaluation of these biomarkers in both preclinical and clinical trial settings will be essential to determine their true utility as early detection tools for NOD individuals at high risk of developing PDAC. To contribute to future research on this topic, we have included a table summarizing the biomarkers proposed to distinguish T2D and T3cD, along with their reported performance metrics (**Table 1**).

**Table 1:** Potential biomarkers and reported metrics of NOD to distinguish PDAC-associated diabetes and T2D.

PDAC-associated diabetes Biomarker vs. T2D	Source	Biological Function/Pathogenesis	AUC/sensitivity & specificity	Reference
Higher serum miR-483-5p, miR-19a, miR-29a, miR-20a, miR-24, and miR-25	Blood	Post-transcriptional regulation of gene expression Unclear role in PDAC-associated diabetes	AUC: 0.902	(102)
Higher galectin-3 Higher S100A9	PDAC cell line proteomics, immune- histochemistry, blood serum levels in patients	Insulin resistance mediators	Galectin-3 AUC: 0.83, 72.1% sensitivity, 86.1% specificity S100A9 AUC: 0.77, 69.8% sensitivity, 58.1 specificity	(103)
Higher adiponectin and IL-1Ra	Blood and serum	Circulating adiponectin correlated with lower subcutaneous adipose tissue and visceral adipose tissue Circulating IL-1Ra correlates with insulin resistance	AUC: 0.91, optimal 83.7% sensitivity, 100% specificity	(104)
Lower Pancreatic polypeptide	Blood	Higher pancreatic polypeptide associated with T2D	Lower incremental AUC	(105)
Glucagon/insulin ratio	PDAC tissue	Higher ratio due to greater area of glucagon expressing cells in islet	At 7.4ng/mU, 77% sensitivity, 69% specificity	(106)
Higher vanin-1 and MMP9	Blood (microarray analysis)	Vanin-1: upstream of cysteamine, key regulator of essential metabolic pathways MMP9: tissue remodeling, tumor weight, and metastasis	AUC: 0.95, 95.83% sensitivity, 76% specificity	(107)
Higher cysteamine, lower GSH, lower PPAR-γ	PDAC cell line	Downstream of Vanin-1, increases oxidative stress leading to islet dysfunction	Cysteamine, GSH, PPAR-γ AUCs: 0.844, 0.863, 0.826	(108)

## A conceptual framework for identifying PDAC-associated diabetes in NOD individuals

**Figure 5:**
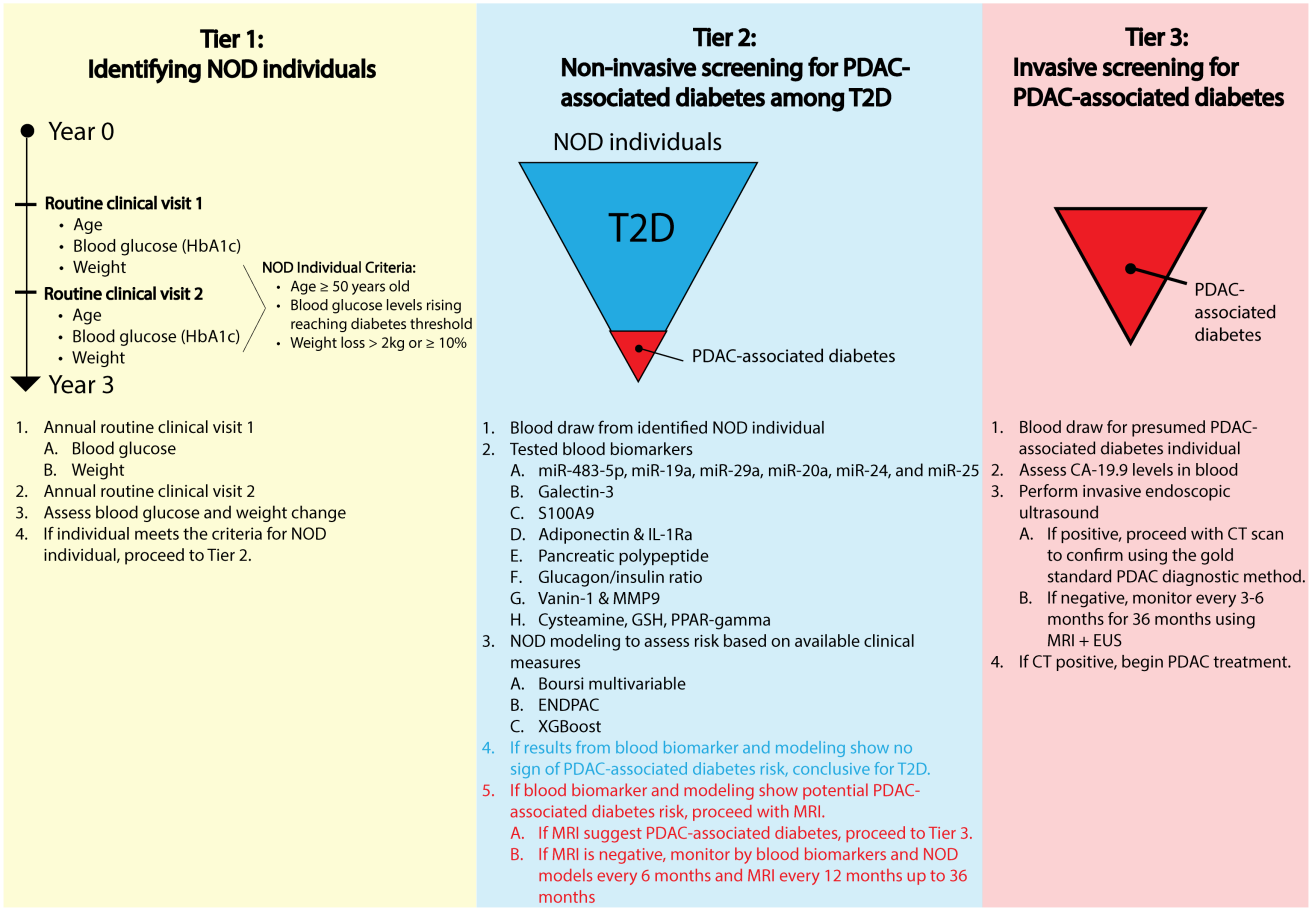
A conceptual framework of a tiered approach to theoretically enrich PDAC-associated diabetes population within the NOD risk group using a multimodal strategy involving clinical presentation, non-invasive screening, PDAC-associated diabetes modeling, and invasive screening for the highest risk individuals.

One of the greatest challenges to overcome in the establishment of NOD as a clinical actionable risk group is to distinguish the small, but clinically important population of PDAC-associated diabetes individuals among the greater proportion of T2D individuals within the NOD group. To address this challenge, we propose a tiered conceptual framework, that begins with the identification of all NOD individuals, followed by non-invasive risk stratification, and ultimately the use of imaging and invasive procedures reserved for those at the highest risk, based on current evidence (**Figure 5**). In tier 1, the goal is to identify individuals with NOD based on routine clinical visits showing evidence for increased blood glucose levels (14–18, 21–27, 90), paired with weight loss equivalent of 2kg or ≥ 10% within the last 3 years in individuals 50 years and older (15, 19, 20, 25, 93). In tier 2, a non-invasive screening strategy is applied to distinguish PDAC-associated diabetes from T2D by evaluating emerging blood biomarkers such as increased miRNAs, galectin-3, S100A9, adiponectin and IL-1Ra, glucagon/insulin ratio, vanin-1 and MPP9, cysteamine, lower GSH, lower PPAR-γ, and lower pancreatic polypeptide paired with an appropriate modeling strategy using the available clinical parameters for risk-assessment such as the Boursi multivariable model, ENDPAC, or machine-learning approaches such as XGBoost, to refine risk assessment (102–108). If the biomarker tests and modeling results from tier 2 are non-indicative of PDAC-associated diabetes, the patient likely has T2D and does not need further clinical assessment. Alternatively, if the results from the tier 2 blood biomarkers and NOD modeling are indicative of PDAC-associated diabetes across a subset of these experimental biomarkers and/or evidenced through modeling, these individuals will proceed to pancreatic imaging with magnetic resonance imaging (MRI). If the MRI shows no indication of PDAC, the individual will be monitored by blood biomarkers and NOD modeling every 6 months and MRI every 12 months up to 36 months extrapolated from current NOD prospective trials and proposed high-risk surveillance recommendations (109–112). In contrast, if MRI reveals features suspicious for PDAC, individuals advance to the tier 3 approach wherein these individuals will be assessed for the standard PDAC diagnostic biomarker, CA-19.9 and undergo EUS. A positive EUS would justify the use of computed tomography (CT) imaging to confirm PDAC diagnosis. On the other hand, if the EUS results are negative, these individuals will be monitored every 3–6 months up to 36 months for clinical signs and symptoms for PDAC.(**Figure 5**) By using a tiered approach to identify PDAC-associated diabetes in the NOD risk group, we would limit costly invasive tools for the highest risk individuals, prevent cumulative radiation exposure by CT (112), while acknowledging that the greatest proportion of NOD individuals will have T2D.

## Ongoing NOD clinical trials

Currently, there are three clinical trials underway to assess the prospective use of monitoring NOD individuals for PDAC development.

**Figure 6:**
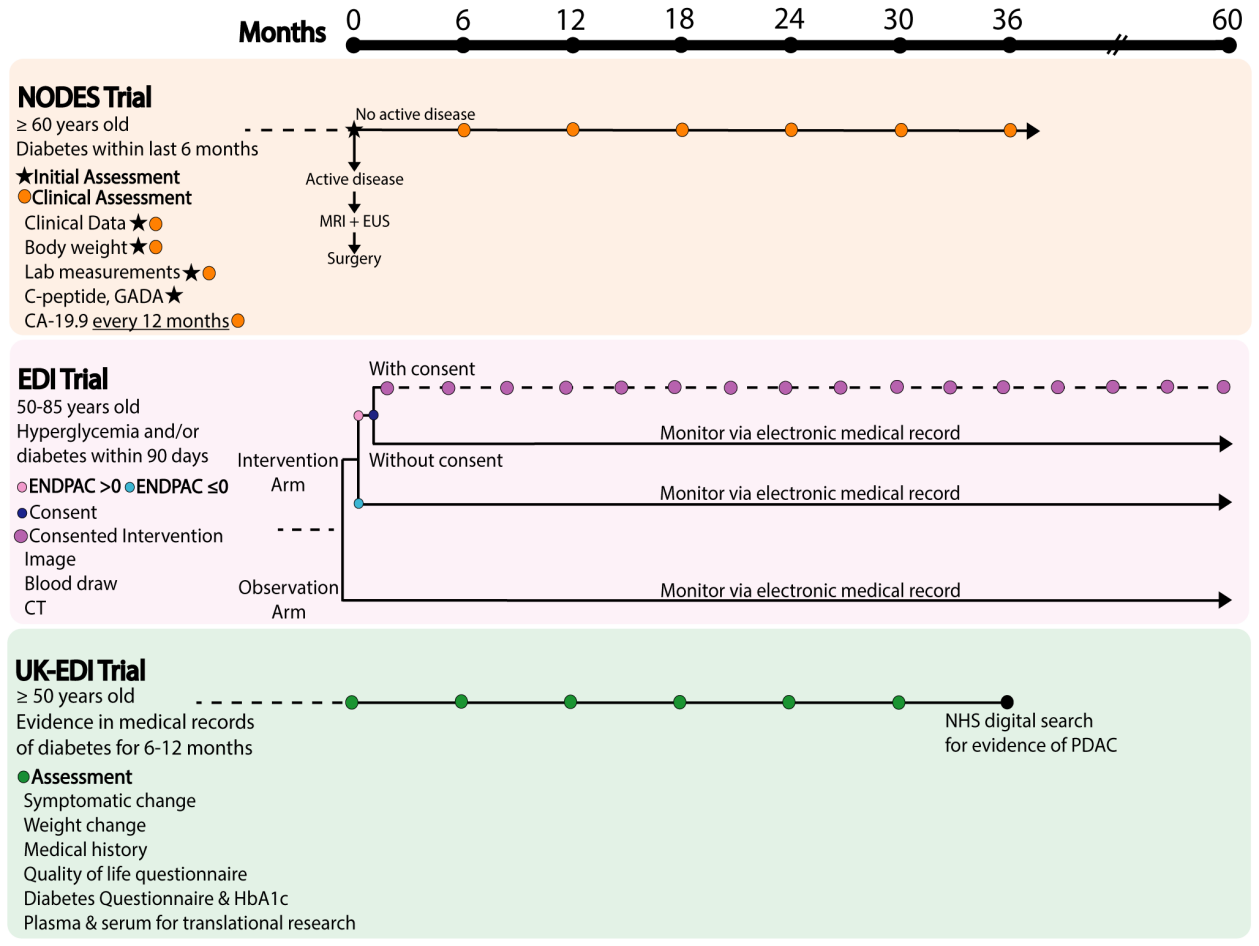
Outline of prospective clinical trials (NODES, EDI, UK-EDI) in NOD individuals using various repeated clinical assessments over time with the primary objective to detect PDAC onset as early as possible.

The NODES trial (NCT04164602) is a prospective study that aims to assess the utility of a screening protocol to identify individuals 60 years of age and older at risk of PDAC development within 6 months of a diabetes diagnosis. Recruited patients will undergo non-invasive clinical assessment for active disease, and body weight change along with a diabetes assessment via C-peptide and glutamic acid decarboxylase antibodies measurements. Furthermore, a fasting blood sample will be drawn for laboratory testing, and an unspecified preclinical metabolomics panel will be validated to help distinguish PDAC-associated T3cD and T2D. If the initial lab test suggests potential concern of active disease, the individual will undergo an MRI or endoscopic ultrasound to determine if the patient requires immediate surgical referral. If not, the patient will undergo clinical assessment every 6 months for 36 months (**Figure 6**).

The Early Detection Initiative (EDI) (NCT03731637, NCT04662879) is a randomized controlled trial where patients between 50 and 85 years of age with the presence of hyperglycemia and/or diabetes within 90 days will be randomized into an observation arm and an intervention arm. Individuals with an ENDPAC score of >0 will be consented to serial blood collection and imaging at baseline and at a 6 months timepoint. The overall objective of this study is to integrate the ENDPAC score, blood collection, and imaging to assess whether this intervention protocol can effectively create a stage shift by reducing the incidence of late-stage (stage III/IV) PDAC diagnoses in NOD populations (**Figure 6**) (109, 110).

Finally, UK-EDI (ISRCTN12805385) is another early detection prospective trial of NOD in the UK that aims to recruit 2500 patients of 50 years and older with NOD to help establish a potential screening regimen for NOD individuals at risk of PDAC development. NOD-qualifying individuals are assessed by their medical history and early clinical symptoms of PDAC over the last 6–12 months, while blood samples are collected to test for proposed differential biomarkers of T2D and PDAC-associated diabetes. Patients are monitored for symptom change, weight change, quality of life, and diabetes management with blood samples drawn to test for HbA1c levels every 6 months for 24 months. Plasma and serum samples are also collected for future translational studies. At 36 months, an NHS Digital search will be conducted to determine the number of PDAC diagnoses within the cohort (111). Collectively, these clinical trials represent exciting developments in the field of PDAC and NOD, and the results may inform future screening protocols to identify patients at the highest risk of PDAC development (**Figure 6**).

## Limitations of NOD as a clinically relevant risk group

Although mounting preclinical and clinical evidence continues to emerge in support of identifying PDAC-associated diabetes within the NOD population as an early detection screening strategy, there remains several outstanding obstacles that hinder the dissemination of NOD as an emerging risk group into clinical practice. Current methods of PDAC early detection screening rely predominantly on traditional imaging methods such as CT, MRI, and EUS that can lead to false-positive or -negative results (113, 114). Specifically, high-risk individuals such as familial pancreatic cancer or inherited genetic cancer syndromes like Peutz-Jeghers syndrome undergoing annual surveillance often develop cancer progression despite no pancreatic lesions detected by imaging. Furthermore, diagnosis of PDAC from monitored lesions in high-risk groups also remain low (115). The success seen in early traditional screening in PDAC have indicated that prevention may not be possible, but early detection may allow tumors to be diagnosed at a lower stage. Continual research in a multimodal approach to PDAC surveillance involving reliable biomarkers will be essential to improve screening and possibly shift screening in PDAC from delayed cancer progression to a curative approach (114).

One of the critical limitations is the low prevalence of this statistically rare group among a highly diagnosed condition such as T2D that continues to pose a challenge in identifying these individuals (14). Currently, there are limitations within the clinical setting in recognizing T3cD and preventing the misdiagnosis of T3cD as T2D. This is partially due to the overlapping nature of the clinical features between T2D and T3cD along with a lack of clarity in the defining criteria for T3cD (56, 57). Growing awareness of T3cD and establishing concrete diagnostic criteria should lead to more accurate diagnoses. To further complicate this matter, T3cD covers a broad range of causes with chronic pancreatitis being the most identified form of T3cD (116). As such, clinical recognition and significance of T3cD have preferentially focused on chronic pancreatitis. At present, without the accurate recognition of T3cD in clinical practice and clearer resolution for differential T3cD causes, we await the continuous investigation of differential biomarkers of T2D, T3cD caused by non-PDAC diseases, and PDAC-associated diabetes in preclinical and clinical studies.

While the highlighted PDAC-associated diabetes biomarkers may theoretically improve the distinction between T2D and PDAC-associated diabetes, the application of a broad panel of PDAC-associated diabetes biomarkers derived from multiple independent cohorts may be unrealistic at this time. With the rarity of PDAC-associated diabetes among NOD individuals and the limited availability of specialized assays across various reference centres, improvement of major national and international collaborations and the establishment of multi-centre consortiums will be the first steps to translate theory into practice (33).

Another limitation within the foundational studies of NOD research that needs to be addressed is the use of various methods of glucose measurement due to choice or as a bottleneck of retrospective data often leading to variability in the measure of diabetes status. This leads to dependency on glycemia data reported at clinical onset instead of glycemic onset, results from questionnaires, Medicare claims, hospital discharge files (25), and medication data instead of glycemia measures using HbA1c or fasting blood glucose (15). Under these circumstances, diabetes may be under-reported and temporal change in glycemia may not be as clear. To mitigate this challenge, prospective studies with clear, consistent, and quantitative glycemia definitions will be essential.

Currently, the Consortium for the study of Chronic Pancreatitis, Diabetes, and Pancreatic Cancer (CPDPC) is establishing a NOD cohort of 10, 000 subjects who are 50 years and older to estimate the 3-year probability of PDAC in NOD individuals, create a biobank of NOD clinical specimen, and to further develop screening protocols that incorporate imaging and clinical algorithms to better identify PDAC-associated diabetes in NOD individuals (117). By establishing this patient cohort, it will help further refine clinical parameters and help establish clearer protocols in a prospective manner.

In our view, a significant gap remains in the clinical knowledge on the effects of glycemia on PDAC patient outcome. Although strong preclinical evidence indicates the important relationship between diabetes and PDAC, there is a lack of attention to the longitudinal glycemia management in PDAC patients. Our group is currently conducting a randomized pilot study called PEGASUS (NCT05132244) at the BC Cancer Agency in Vancouver, Canada, to investigate the interplay between diabetes and PDAC by closely monitoring glucose levels in PDAC patients and associating its effect on clinical outcome (118). This information could provide the translational evidence that diabetes is a critical player in PDAC patient outcome that could further corroborate the importance of monitoring diabetes, a manageable condition, in the precancer, early detection setting.

## Discussion

Given the growing global burden of PDAC, it is fundamental to identify early signs of PDAC development to enable intervention with treatment earlier in the disease course. We have highlighted PDAC-associated diabetes among NOD individuals, as a key subgroup of patients that have an increased risk of PDAC development that should be further considered in the early detection setting. Although evidence suggests an eight times higher risk in PDAC-associated diabetes in NOD individuals, the percentage of these individuals among the highly diagnosed T2D is between 0.25-3% shown in the studies discussed. Therefore, this emerging risk group currently lacks clinical application and its success in becoming a risk group is contingent on further knowledge development in diabetes, PDAC, and the interplay between these two diseases. In this review, we highlighted distinctions of each type of diabetes to anchor NOD as a terminology defined as a new diabetes diagnosis that theoretically can be T1D, T2D or any forms of T3cD. However, NOD has transitioned from a terminology to an emerging PDAC risk group that requires careful delineation of various forms of diabetes, particularly T2D, T3cD originating from CP, and PDAC-associated diabetes.

To translate this risk group into clinical practice, there are many hurdles to overcome. Most importantly, PDAC-associated diabetes must be disentangled from T2D, while balancing accurate detection, cost-effectiveness of screening, and nonmaleficence. As such, we have proposed a conceptual framework for a tiered screening strategy focused on non-invasive screening for all NOD individuals that progressively applies more specific and invasive tests to individuals deemed highest risk among NOD individuals to help identify the PDAC-associated diabetes population. This strategy adopts concepts from the current literature highlighted in this review and merges the currently established clinical parameters of NOD individuals, preclinical non-invasive blood-based biomarkers distinguishing PDAC-associated diabetes and T2D, NOD risk-assessment modeling, and imaging and invasive procedures. Even though this conceptual framework is hypothetical with no intent to inform clinical practice, it acts to integrate existing knowledge into a single structure to highlight key tools in the development of NOD as an emerging PDAC risk group in the early detection setting.

To clinically implement NOD as a risk group, we are reliant on the success of clinical trials. We highlighted multiple ongoing clinical trials that have established their own protocols to prospectively further our understanding of PDAC-associated diabetes within the NOD population, determine clinical parameters and biomarkers enriched in PDAC-associated diabetes, and assess the clinical effectiveness of screening NOD individuals in the early detection setting.

At this time, the ongoing limitations within the field are a lack of T3cD recognition, established clinical criteria for T3cD, consensus on biomarkers associated with PDAC-associated diabetes, and consistent methods of blood glucose measurements across retrospective studies. However, these issues can be mitigated by advancements and additional insight from prospective studies and biomarker discovery for PDAC-associated diabetes.

With rising cases of diabetes and PDAC, and the strong interconnection between these two diseases, there is a continuous public health need to improve the evaluation of NOD patients for PDAC. NOD studies have shown that a proportion of PDAC patients will show early signs of cancer through diabetes presentation months to years before their cancer diagnosis, representing an untapped opportunity to use blood glucose levels in the early detection setting of PDAC. Although NOD as a risk factor for PDAC has been known for years, the evaluation of NOD in the general population as a clinically actionable PDAC risk group has not realized its potential due to the complex, individually distinct nature of PDAC, diabetes, and NOD. As a low incidence disease (33), PDAC high-risk groups make up an even smaller proportion of all PDAC cases with surveillance programmes requiring more evidence for its utility in PDAC survival outcomes (119). In accordance with this, imaging techniques for PDAC are in need of improvement to accurately capture PDAC at its earliest presentation in both PDAC-associated diabetes individuals as well as other established PDAC high-risk groups (119). Meanwhile, the NHS Health Check has shown that a lack of annual diabetes risk assessment, exclusive HbA1c testing only in high-risk individuals, and the exclusion of individuals age 74 and older for testing contribute to the lack of recognition of pre-surgical PDAC-associated diabetes (120). With NOD being a high incidence diagnosis with many proposed preclinical biomarkers and models to help distinguish PDAC-associated diabetes from T2D, there is a strong need to validate these biomarkers and models in multicentre studies to reach consensus on the recommended guidelines for enriching PDAC-associated diabetes within NOD individuals (33, 43, 119). While it is clear that future efforts are needed to better establish this emerging risk group, the pursuit of the ongoing research strategies has the potential to improve the prognosis of PDAC, to further disentangle the role of diabetes in PDAC patients, and to ultimately combat this disease at its earliest presentation.
